# The cAMP-Dependent Protein Kinase Inhibitor H-89 Attenuates the Bioluminescence Signal Produced by *Renilla* Luciferase

**DOI:** 10.1371/journal.pone.0005642

**Published:** 2009-05-21

**Authors:** Katie J. Herbst, Michael D. Allen, Jin Zhang

**Affiliations:** 1 Department of Pharmacology and Molecular Sciences, The Johns Hopkins University School of Medicine, Baltimore, Maryland, United States of America; 2 The Solomon H. Snyder Department of Neuroscience and Department of Oncology, The Johns Hopkins University School of Medicine, Baltimore, Maryland, United States of America; Newcastle University, United Kingdom

## Abstract

**Background:**

Investigations into the regulation and functional roles of kinases such as cAMP-dependent protein kinase (PKA) increasingly rely on cellular assays. Currently, there are a number of bioluminescence-based assays, for example reporter gene assays, that allow the study of the regulation, activity, and functional effects of PKA in the cellular context. Additionally there are continuing efforts to engineer improved biosensors that are capable of detecting real-time PKA signaling dynamics in cells. These cell-based assays are often utilized to test the involvement of PKA-dependent processes by using H-89, a reversible competitive inhibitor of PKA.

**Principal Findings:**

We present here data to show that H-89, in addition to being a competitive PKA inhibitor, attenuates the bioluminescence signal produced by *Renilla* luciferase (RLuc) variants in a population of cells and also in single cells. Using 10 µM of luciferase substrate and 10 µM H-89, we observed that the signal from RLuc and RLuc8, an eight-point mutation variant of RLuc, in cells was reduced to 50% (±15%) and 54% (±14%) of controls exposed to the vehicle alone, respectively. *In vitro*, we showed that H-89 decreased the RLuc8 bioluminescence signal but did not compete with coelenterazine-h for the RLuc8 active site, and also did not affect the activity of Firefly luciferase. By contrast, another competitive inhibitor of PKA, KT5720, did not affect the activity of RLuc8.

**Significance:**

The identification and characterization of the adverse effect of H-89 on RLuc signal will help deconvolute data previously generated from RLuc-based assays looking at the functional effects of PKA signaling. In addition, for the current application and future development of bioluminscence assays, KT5720 is identified as a more suitable PKA inhibitor to be used in conjunction with RLuc-based assays. These principal findings also provide an important lesson to fully consider all of the potential effects of experimental conditions on a cell-based assay readout before drawing conclusions from the data.

## Introduction

Protein kinases control many intracellular signaling cascades by enzymatically transferring the γ-phosphate of ATP to amino acid side chains of protein targets. Aberrant signal transduction, such as dysregulation of protein kinases, can result in pathophysiological states [1]. As many signal transduction cascades have shared molecular components, it is fundamentally important to study protein kinases in cells where the entire signaling network remains intact [2]. Many cell-based assays have focused on determining the dependence of a specific cellular effect on a given kinase over a set period of time [3]–[5]. More recently, however, there have been efforts to design biosensors which are capable of monitoring signaling dynamics in real-time [5]–[8]. These newer tools have the potential to elucidate the dynamic series of molecular interactions and modifications that contribute to a specific cellular effect. Together, the application of current and the development of new cell-based assays for kinase activity will continue to provide insights regarding the connection between the regulation and dynamics of kinase activity and a given functional response.

Cyclic AMP-dependent protein kinase (PKA), one of the first discovered protein kinases, is well characterized [9]. PKA plays a role in, among other things, transcriptional control of genes downstream of the cAMP response element (CRE) [10], maintenance and control of several metabolic processes, rearrangement of actin for muscle contraction and relaxation [11], and DNA replication [12]. It is also implicated in a number of diseases such as Alzheimer's disease [13], cancer [14], heart disease [15], and diabetes [16]. Consequently, PKA remains one of the most frequently studied protein kinases [9]. One commonly used technique that serves to monitor the functional effects of PKA in cells is bioluminescence. Bioluminescence is an endogenous characteristic of many organisms in which an enzyme (luciferase) oxidizes a substrate (luciferin) and emits photons. Scientists frequently take advantage of luciferase enzymes to engineer a variety of bioassays suited for studies *in vitro*, in cell-based assays, and *in vivo*. Bioluminescence-based assays are desirable because they provide a low background, are simple to use, and are non-destructive when used in living systems [4], [6].

The most frequently used luciferase enzymes in bioassays are from the firefly *Photinus pyralis* (FLuc) and the sea pansy *Renilla reniformis* (RLuc). FLuc is 62kDa, ATP-dependent, and emits light at 560nm, whereas RLuc is 36kDa, ATP-independent, and emits at 480nm [5]. These different properties often determine for which type of assay each luciferase would be better suited [3]. A common application of these proteins is for reporter gene assays that detect levels of transcription in a cell. In this case, the cDNA for the luciferase is fused downstream of a given response element. In response to various stimulations, the response element is activated to a level which correlates with the amount of luciferase, and thus the signal, produced. A common control for these reporter gene assays is to have a spectrally distinct reporter gene under the control of a highly active promoter to serve as a transfection control or as a control for cell viability. Therefore FLuc and RLuc are commonly used concurrently in reporter gene assays to test the dependence of specific stimuli on transcription levels in cells.

In addition to reporter gene assays, bioluminescence proteins are being used in the design of biosensors that can capture signaling dynamics in living cells. Such live-cell tools have been developed to detect protein-protein interactions, second messenger dynamics, enzyme (namely protease and kinase) activity, and receptor activation by utilizing techniques such as luciferase complementation assays, bioluminescence resonance energy transfer (BRET), and circular permutation of luciferases, [6]–[8]. When developing such assays to specifically monitor kinase activity, PKA often serves as a prototype for the design of new biosensors.

Cellular bioluminescence-based assays provide readout for a specific cellular event such as gene transcription. Testing the dependence of a specific cellular response on a molecule of interest is achieved via pharmacological stimulation or inhibition of a protein of interest. In the case of PKA, commonly used activators are agonists of the β-adrenergic receptors or activators of adenylyl cyclases, both of which are upstream activators of PKA. To inhibit PKA activity in cells, the reversible and competitive inhibitor of PKA, H-89 (N-[2-(p-bromocinnamylamino)ethyl]-5-isoquinolinesulfonamide) is most commonly used [17]. Generally, stimulation or inhibition of a protein of interest provides direct evidence regarding the protein's role in the cellular process under study. Sometimes, however, it is possible that an agent that is added to the experiment to inhibit a specific target is actually directly modulating the activity of the luciferase.

We encountered this scenario while characterizing a novel cell-based assay to detect PKA activity using a more stable and brighter version of RLuc (RLuc8) [18] as the reporting unit. Upon addition of H-89 to cells, we noticed a rapid, significant decrease in RLuc8 signal in our PKA-independent negative control (unpublished), and we thus suspected that the decrease in RLuc8 signal was not due to PKA inhibition alone. We further investigated the nature of the decrease in RLuc8 signal and showed that H-89 was responsible for the attenuation of the bioluminescence signal.

## Results

**Figure 1:**
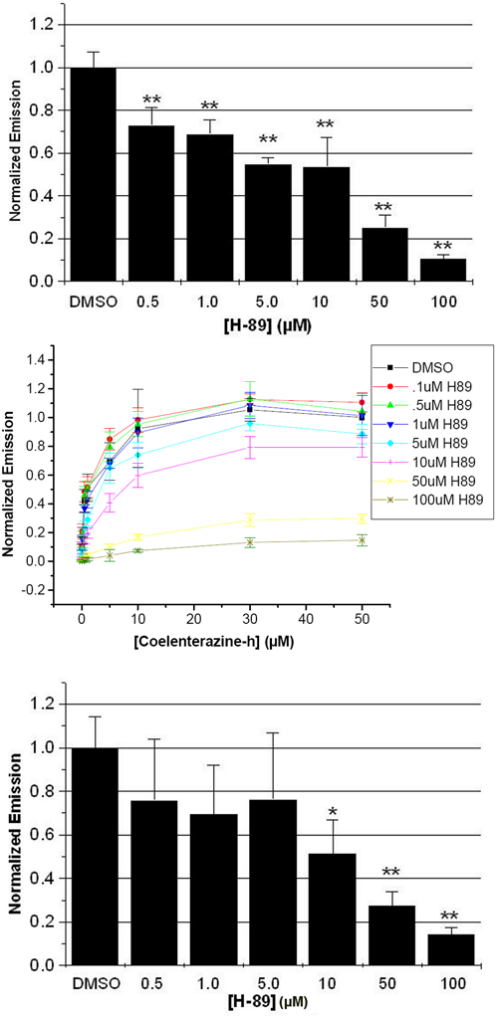
H-89 decreases the bioluminescence signal produced by RLuc variants. (A) In a 96-well plate, HEK293T cells expressing RLuc8 and PKIα were preincubated for 10 minutes with various doses of H-89 (n = 4 for each dose of H-89). 10 µM coelenterazine-h was added to each sample and luminescence was detected immediately after addition. **: p<0.005 (compared to DMSO). (B) 1 nM RLuc8 was preincubated for 10 minutes over a range of concentrations of H-89 (n = 3 for each dose of H-89) and luminescence was detected immediately after coelenterazine-h addition. (C) In a 96-well plate, HEK293T cells expressing RLuc and PKIα were preincubated for 10 minutes with various doses of H-89 (n = 3 for each dose of H-89). After addition of 10 µM coelenterazine-h, luminescence was detected immediately. * p<0.05, ** p<0.005.

We hypothesized that there were two possibilities for the decrease in RLuc8 signal after addition of H-89: PKA modulates RLuc8 activity in such a way that PKA inhibition decreases the signal from RLuc8, or H-89 directly attenuates the signal produced by RLuc8. In order to test the possibility that H-89 directly attenuated the RLuc8 bioluminescence signal while avoiding the complication of PKA-dependent inhibition of RLuc8, we expressed RLuc8 in HEK293T cells along with the PKA peptide inhibitor (PKIα) [19], as this would ensure that PKA was inactive [20], [21]. With PKA inhibited, we could directly monitor the activity of RLuc8 independent of any PKA effect. The transfected cells were plated into 96-well plates and allowed to touch down for 24 hours. After a ten minute incubation with concentrations of H-89 ranging from 0.5–100 µM, total light output was measured. In this cellular context with PKA inhibited by PKIα, after immediate addition of coelenterazine-h (a substrate for luciferases from the *Renilla* family) we noticed that the signal from RLuc8 decreased at concentrations of H-89 as low as 0.5 µM. Notably, at 10 µM H-89, the concentration used in most cell-based studies to inhibit PKA activity, the RLuc8 signal was reduced to 54% (±14%) of control receiving vehicle alone (Fig. 1A). In cells expressing RLuc8 with active PKA, in other words without overexpression of PKIα, a similar decrease in signal was observed (Fig. S1). Together, these data suggested that H-89, and not PKA, was responsible for abating the bioluminescence signal produced by RLuc8 oxidation of coelenterazine-h.

To confirm that H-89 was responsible for the reduction in bioluminescence signal, we tested the effect of H-89 on RLuc8 *in vitro*. Purified RLuc8 was added to each well of a 96-well plate at 1 nM and incubated in Hank's balanced salt solution (HBSS) supplemented with concentrations of H-89 ranging from 0.1–100 µM for 10 minutes. Coelenterazine-h was then added over a range of concentrations from 0.1–50 µM and signal was detected immediately after addition. By plotting the rate of coelenterazine-h oxidation (normalized emission) versus concentration of coelenterazine-h, it was evident that increasing concentrations of H-89 decreased the maximal velocity (Vmax) of the reaction, suggesting both that H-89 does not compete with coelenterazine-h in the RLuc8 active site, and that the signal cannot be restored by adding more coelenterazine-h to the reaction (Fig. 1B). Importantly, at 10 µM coelenterazine-h, a commonly used concentration of substrate for *Renilla* luciferases, the IC_50_ of H-89 on RLuc8 is 21.0 µM (±4.0 µM) which is just twice the most commonly used dose in cell-based assays for inhibition of PKA. Therefore, using the most common doses of coelenterazine-h and H-89 in an RLuc8-based assay for PKA will result in a significant reduction in RLuc8 signal that could be wrongfully attributed to a decrease in PKA activity.

Since RLuc8 is a relatively new variant of RLuc, and because most current bioassays utilizing *Renilla* luciferases are based on RLuc, we wanted to test the effect of H-89 on RLuc in cells. Thus we co-expressed RLuc and PKIα in HEK293T cells and detected bioluminescence in a multi-well format. After a 10 minute preincubation with H-89, we observed that H-89 decreased the signal of RLuc in a similar fashion to RLuc8. Specifically, with 10 µM H-89, RLuc signal was abated to 51% (±15%) of control receiving vehicle alone (Fig. 1C), and we observed a similar pattern of RLuc signal attenuation in cells that expressed RLuc without PKIα (Fig. S2). The effect of H-89 on both RLuc8 and RLuc has two significant implications. First, H-89 should be used with caution in RLuc- or RLuc8-based assays or alternative inhibitors or assays should be used. Secondly any data previously generated from such assays may have an element of bias.

**Figure 2:**
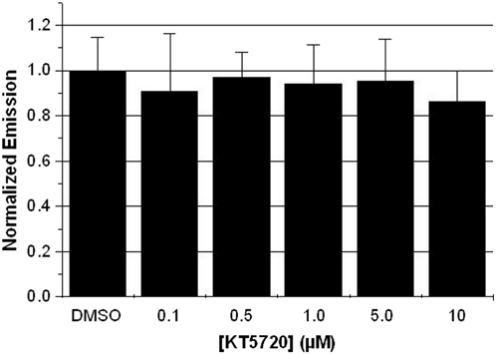
KT5720 does not reduce the bioluminescence signal from RLuc8 in cells. In a 96-well plate, HEK293T cells expressing RLuc8 and PKIα were preincubated with KT5720 (n = 3 for each dose of KT5720). No reduction of signal was observed immediately after addition of 10 µM coelenterazine-h.

In order to study the functional dependence of PKA in a given cellular system, it is important to have a reversible inhibitor of PKA that can be used in cells. For this reason, and for the purpose of our studies, we wanted to find a reversible PKA inhibitor that could be used in cells and not inhibit RLuc8 activity. Thus, we tested the activity of RLuc8 in the presence of the small molecule PKA inhibitor KT5720 [22]. Again, we transfected RLuc8 and PKIα into HEK293T cells. In a multi-well format, we allowed the cells to preincubate for 20 minutes with the reversible PKA inhibitor KT5720 with concentrations ranging from 0.1–10 µM as this spans those commonly used in cell based-assays [23]. After addition of coelenterazine-h, we observed that KT5720 did not modulate RLuc8 activity across the tested range of concentrations (Fig. 2). Thus, KT5720 can be used as an alternative to H-89 to inhibit PKA activity in RLuc8-based bioassays.

**Figure 3:**
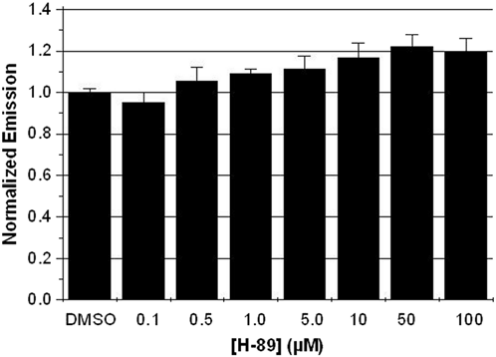
H-89 does not abate the bioluminescence signal produced by FLuc in vitro. 1 nM FLuc was preincubated with various doses H-89 (n = 4 for each dose of H-89). After D-luciferin addition, reduction in signal was not observed with any of the treatments.

Similarly, we wanted to test the effect of H-89 on FLuc activity. Since FLuc and RLuc are not homologous we predicted that H-89 would not necessarily be an inhibitor of FLuc. Also, if H-89 did not inhibit FLuc activity, FLuc would be suitable for use in luciferase-based PKA assays. In a 96-well plate, 1 nM purified FLuc was preincubated with H-89 over the same range of concentrations, 0.5–100 µM, which we used in previous experiments. Luminescence was recorded immediately after D-luciferin (the substrate for FLuc) addition and it was found that H-89 showed no decrease in FLuc signal *in vitro* (Fig. 3); it was also shown that FLuc activity was not reduced by 10 µM KT5720 (Fig. S3). Therefore, FLuc can be used in the presence of H-89 or KT5720 and would be suitable as a readout for PKA bioassays.

**Figure 4:**
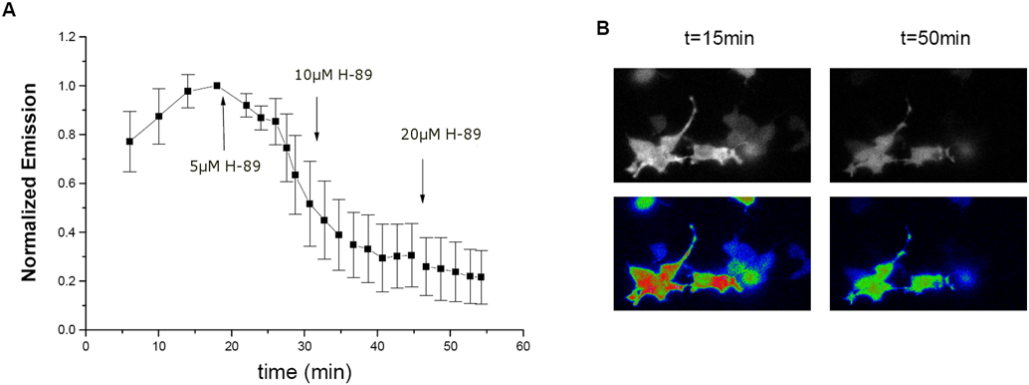
H-89 reduces the signal from RLuc8 in single cells. HEK293T cells (n = 7) expressing RLuc8 and PKIα were imaged in the presence of 10 µM coelenterazine-h. (A) After addition of 5 µM H-89, RLuc8 signal decreases. Additional doses of H-89 decrease the signal further. (B) Channel intensity images (top) and pseudocolor images (bottom) of cells corresponding to (A) at t = 15 min (before H-89 addition) and t = 50 min (when signal has reached maximum inhibition).

Cell-based assays offer the advantage of detecting single-cell behaviors and at the same time present the challenge of dealing with cell-cell variations. To confirm the inhibitory effects of H-89 on RLuc variants obtained with populations of cells in the plate-reader format and characterize cell-cell variations, we imaged cells expressing RLuc8 and PKIα on a microscope. Coelenterazine-h was added to cells and allowed to incubate for 10 minutes. Images were acquired at 4 minute intervals from a population of seven cells until a two-point steady baseline was achieved. Then, 5 µM H-89 was added to the imaging dish resulting in an initial decrease in RLuc8 signal. After 10 minutes, the RLuc8 signal was reduced to 51% (±17%) of the maximal value, consistent with that obtained from the larger population of cells in the multi-well format (Fig. S4). Over another 25 minute period two additional doses of H-89 decreased the RLuc8 emission to 20% (±10%) of peak intensity (Fig. 4). This data shows that though the inhibition of RLuc8 by H-89 takes a period of 20 minutes to achieve maximal inhibition, the effects of inhibition can be seen immediately after addition. Thus, a decrease in luciferase signal will be observed after H-89 addition regardless of incubation time. A more substantial reduction of signal, however, will be observed in assays that use longer H-89 incubation periods.

The single cell luciferase experiments provide two other key observations. First, it is clear by looking at the initial images (t = 15) that the cells have a normal appearance, demonstrating that overexpression of neither Rluc8 nor PKIα is toxic to the cells (Fig. 4B). Further, since there are no morphological changes to the cells during the imaging timeframe, the attenuation of bioluminescence signal upon H-89 addition cannot be attributed to a cell shape change.

## Discussion

Protein kinases serve as the primary regulators of signal transduction cascades by phosphorylating protein targets in response to various extracellular and intracellular stimuli. In turn, this phosphorylation contributes to the propagation of the signal until a desired physiological response is generated. PKA is an example of a ubiquitously expressed protein kinase that is involved in a plethora of vital cellular functions. Elucidation of many of the functional roles of PKA in regulating various cellular processes relies on bioluminescence-based cellular assays. However, we present evidence that H-89, a commonly used PKA inhibitor, can directly decrease the bioluminescence signal resulting from RLuc activity.

Though there are a few examples of small molecules that act as inhibitors of FLuc reported in literature [24]–[26], one group at the NIH Chemical Genomics Center hypothesized that often times such molecules are overlooked. After performing a quantitative high throughput screen of over 70,000 compounds, they identified 2311 inhibitors for FLuc. Many of these inhibitors were shown to be competitive inhibitors of FLuc and the activity could be fully restored by increasing the concentration of luciferin or ATP [27]. Most of these same compounds, however, were not potent inhibitors of RLuc activity, likely because RLuc is an ATP-independent enzyme. Though there has been an effort to identify classes of drugs with common structures that act as luciferase inhibitors, it remains important to fully characterize the effect of experimental conditions on the luciferase while utilizing or developing bioluminescence assays. In a recent example, incomplete understanding of the direct effect of a small molecule on a reporter protein led to miss-interpretation of data from a cell-based FLuc assay [28].

Since the mechanism of coelenterazine oxidation by RLuc is not entirely understood, it is difficult to speculate on a mechanism of H-89-induced attenuation of RLuc signal. However, it is known that RLuc shares a conserved catalytic triad and 42% identity with bacterial haloalkane dehalogenases of the LinB family [29]. These proteins utilize the characteristic α/β hydrolase motif to catalytically hydrolyze carbon-halogen bonds [30]. Therefore, we considered the possibility that H-89, a brominated small molecule, could bind to RLuc and induce a conformational change in the enzyme that slows down the oxidation of coelenterazine-h. However, in this case, a brominated small molecule would bind to the RLuc active site and display competitive, not non-competitive, inhibitory properties. Alternatively, H-89 may interact with coelenterazine-h and affect its properties. Indeed, we observed that the presence of H-89 caused a change in the fluorescence spectrum of coelenterazine-h in buffer (Fig. S5), suggesting some interactions between these two molecules, although no covalent adduct was observed via mass spectrometry (Fig. S6).

Here we present evidence that the PKA inhibitor H-89 attenuates the bioluminescence signal produced by RLuc variants both in cells and *in vitro*. This is an important piece of information which serves two purposes. First, it can help deconvolute data previously generated from RLuc-based assays that looked at PKA-dependent cellular processes as it is likely that the interpretation of such data was inadvertently distorted if H-89 was used. Second, this data will guide the application of current RLuc-based assays for PKA activity and also the development of new bioluminescence-based kinase biosensors. Specifically, we suggest that when utilizing or developing RLuc-based assays for PKA activity, H-89 not be used to test the dependence of the assay on PKA as the data would be unintentionally skewed. As an alternative to H-89, one could use the reversible PKA inhibitor KT5720 or the irreversible peptide inhibitor PKIα, to test the involvement of PKA. Additionally, H-89 can be used in FLuc-based cellular assays without the concern of a non-specific decrease in signal. Overall, this data provides an important lesson to characterize all of the potential effects of the experimental conditions on cell-based assays with proper controls before drawing conclusions from data. Otherwise, a compound or condition that specifically modulates the activity of the reporting unit and not the event under study could influence the interpretation of data.

## Materials and Methods

### Gene construction and protein purification

RLuc8, RLuc, and PKIα were PCR amplified and ligated into pCDNA3 (Invitrogen) for mammalian expression and RLuc8 was ligated into pRSETB (Invitrogen) for bacterial expression. RLuc8 in PRSETB was transformed into BL21(DE3) *E. coli* cells. A single colony was grown to an O.D._600_ of 0.6 and induced with 0.1 mM IPTG. Cells were grown 12 hours at 37°C, spun at 10,000g for 10 min, and then lysed by sonication in lysis buffer (50 mM Tris–HCl, pH 7.4, 300 mM NaCl, 0.2% Triton X-100, and protease inhibitor cocktail (Roche)). Lysed cells were spun at 15,000 rpm for 20 minutes and 100 µL of Ni-NTA beads (Qiagen) was added to the supernatant. The protein/bead mixture was added to a column with a polyethylene disc (Kontes) used as the filter. The column was washed twice in buffer containing 50 mM Tris–HCl, pH 7.4, 300 mM NaCl, and 10 mM imidazole, and then protein was eluted off of the beads via an elution buffer containing 100 mM imidazole. Protein concentration was determined by BCA assay (Thermo Scientific).

### HEK293T-based assays

HEK293T were grown in DMEM cell culture media (Gibco) supplemented with 10% FBS at 37°C with 5% CO_2_. Cells were transfected via calcium phosphate at 60% confluency with RLuc8 or RLuc and PKIα. For plate reader studies, after a 24 hour transfection period, cells were plated at 150,000 cells/well into white-walled, clear- bottom 96 well plates (Corning) coated with 0.1mg/ml poly-D-lysine. After 24 hours, media was replaced with HBSS supplemented with H-89 (Sigma) or KT5720 (Sigma). Benzyl-coelenterazine (Nanolight Technology) was added to a final concentration of 10 µM and luminescence readings were recorded immediately. For imaging, cells were transfected directly into 35 mm imaging dishes and imaged 24 hours later.

### In vitro assays

1 nM RLuc8 or 1 nM FLuc (Promega) was added to white-walled, clear- bottom 96 well plates in HBSS. H-89 was added directly to wells and incubated at 4°C for 10 minutes. Luciferase substrate (Benzyl-coelenterazine for RLuc8 or D-luciferin for FLuc) was added to a final concentration of 10 µM and luminescence was recorded immediately.

### Plate reader luminescence detection

All luminescence readings were obtained on a FLUOstar OPTIMA microplate reader without an emission filter.

### Bioluminescence imaging and analysis

Cells were imaged on a Zeiss Axiovert 200M microscope with a Hamamatsu ImagEM cooled charge-coupled device camera controlled by METAFLUOR software (Universal Imaging, Downingtown, PA). Using a 475DF40 emission filter and 450DRLP dichroic mirror, images were acquired immediately after coelenterazine-h addition. Acquisition time was 30 seconds and images were acquired every 2–4 minutes. H-89 was added directly to the imaging dish. Images were background corrected and processed on Image J software.

## Supporting Information

Figure S1 H-89 attenuates the activity of RLuc8 in cells. RLuc8 was transfected into HEK293T cells and cells were plated in a multiwell format. 24 hours later, cells were pre-incubated with H-89 for 10 minutes. Luminescence was detected immediately after addition of 10 µM coelenterazine-h. H-89 attenuated the activity of RLuc8 in a dose-dependent manner (n = 3). (** p<0.01 compared to DMSO).
(0.04 MB TIF)

Figure S2 H-89 attenuates the activity of RLuc in cells. RLuc was transfected into HEK293T cells. In a multiwell format, cells were pre-incubated with H-89 for 10 minutes. Luminescence was detected immediately after addition of 10 µM coelenterazine-h. H-89 attenuated the activity of RLuc in a dose-dependent manner (n = 3). (** p<0.01 compared to DMSO)
(0.04 MB TIF)

Figure S3 KT5720 does not attenuate FLuc activity in vitro. 10 nM FLuc was pre-incubated with 10 µM KT5720 for 10min. Immediately after D-luciferin addition, there was no attenuation of FLuc activity (n = 5).
(0.03 MB TIF)

Figure S4 Time course of vehicle and H-89 treatments from a population of cells. HEK293T cells expressing RLuc8 and PKIα were incubated in HBSS supplemented with 10 µM coelenterazine-h for 10 minutes and then were treated with 5 µM H-89 or vehicle. There was a rapid, initial decrease in signal upon H-89 addition. Subsequent doses of H-89 further decrease the signal. The signal from the vehicle control cells also decreases due to a combined effect of enzymatic coelenterazine-h oxidation and product (coelenteramide) inhibition of RLuc8. The study supplements that of the single cell experiments (n = 3).
(0.04 MB TIF)

Figure S5 H-89 shifts the emission spectrum of coelenterazine-h. The emission spectra of 10 µM coelenterazine-h in HBSS excited at 280 nM. When compared to vehicle control, 10 µM H-89 changes the emission spectrum.
(0.03 MB TIF)

Figure S6 H-89 and coelenterazine-h do not form a covalent adduct. All samples were diluted in 50% ACN, 0.1% FA, loaded into electrospray needle, sprayed at 900 V, and detected from m/z between 350–1200. A) spectra of H-89. B) Spectra of coelenterazine-h. C) spectra of 1:1, H-89:coelenterazine-h
(0.04 MB TIF)
