# Supplementary figures and images for: The cAMP-Dependent Protein Kinase Inhibitor H-89 Attenuates the Bioluminescence Signal Produced by Renilla Luciferase
Source: PLoS One. 2009 May 21;4(5):e5642. doi: 10.1371/journal.pone.0005642 (PMC2680982; doi:10.1371/journal.pone.0005642)

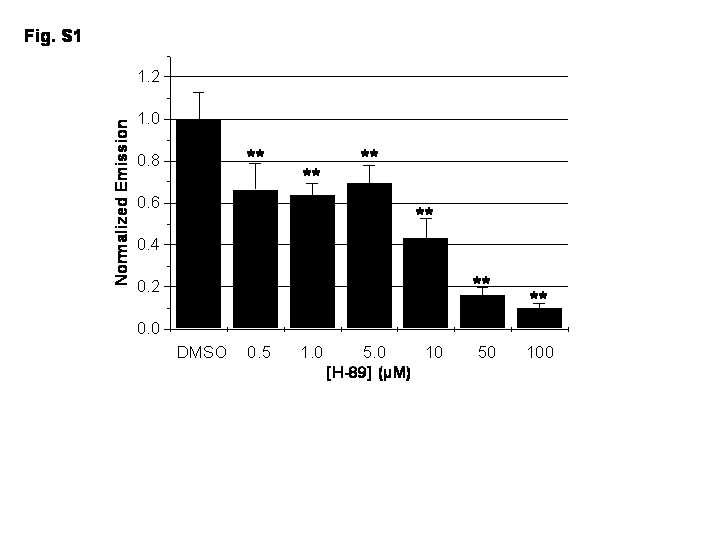

Supplement: Figure S1 — H-89 attenuates the activity of RLuc8 in cells. RLuc8 was transfected into HEK293T cells and cells were plated in a multiwell format. 24 hours later, cells were pre-incubated with H-89 for 10 minutes. Luminescence was detected immediately after addition of 10 µM coelenterazine-h. H-89 attenuated the activity of RLuc8 in a dose-dependent manner (n = 3). (** p<0.01 compared to DMSO). (0.04 MB TIF) [file pone.0005642.s001.tif]

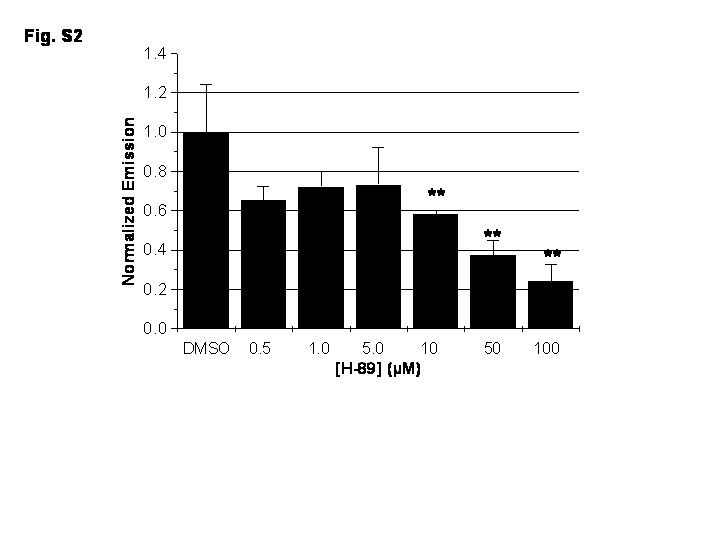

Supplement: Figure S2 — H-89 attenuates the activity of RLuc in cells. RLuc was transfected into HEK293T cells. In a multiwell format, cells were pre-incubated with H-89 for 10 minutes. Luminescence was detected immediately after addition of 10 µM coelenterazine-h. H-89 attenuated the activity of RLuc in a dose-dependent manner (n = 3). (** p<0.01 compared to DMSO) (0.04 MB TIF) [file pone.0005642.s002.tif]

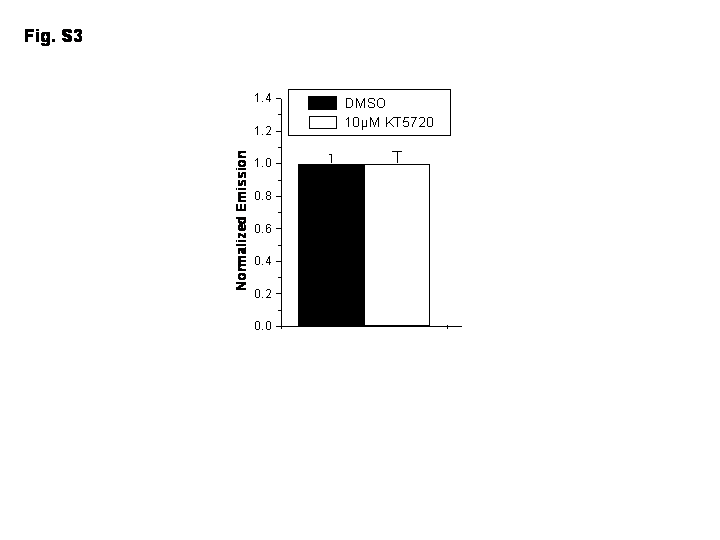

Supplement: Figure S3 — KT5720 does not attenuate FLuc activity in vitro. 10 nM FLuc was pre-incubated with 10 µM KT5720 for 10min. Immediately after D-luciferin addition, there was no attenuation of FLuc activity (n = 5). (0.03 MB TIF) [file pone.0005642.s003.tif]

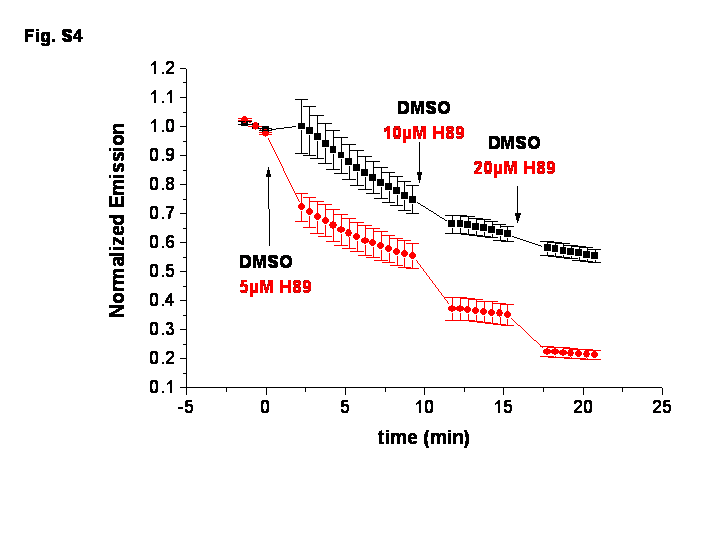

Supplement: Figure S4 — Time course of vehicle and H-89 treatments from a population of cells. HEK293T cells expressing RLuc8 and PKIα were incubated in HBSS supplemented with 10 µM coelenterazine-h for 10 minutes and then were treated with 5 µM H-89 or vehicle. There was a rapid, initial decrease in signal upon H-89 addition. Subsequent doses of H-89 further decrease the signal. The signal from the vehicle control cells also decreases due to a combined effect of enzymatic coelenterazine-h oxidation and product (coelenteramide) inhibition of RLuc8. The study supplements that of the single cell experiments (n = 3). (0.04 MB TIF) [file pone.0005642.s004.tif]

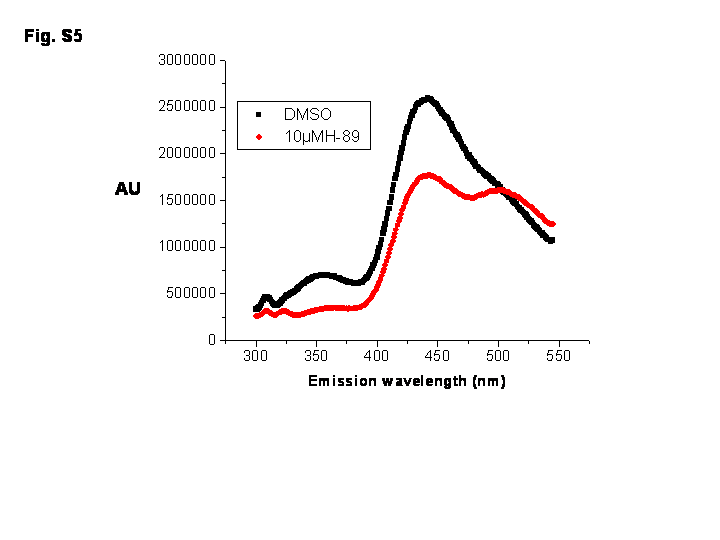

Supplement: Figure S5 — H-89 shifts the emission spectrum of coelenterazine-h. The emission spectra of 10 µM coelenterazine-h in HBSS excited at 280 nM. When compared to vehicle control, 10 µM H-89 changes the emission spectrum. (0.03 MB TIF) [file pone.0005642.s005.tif]

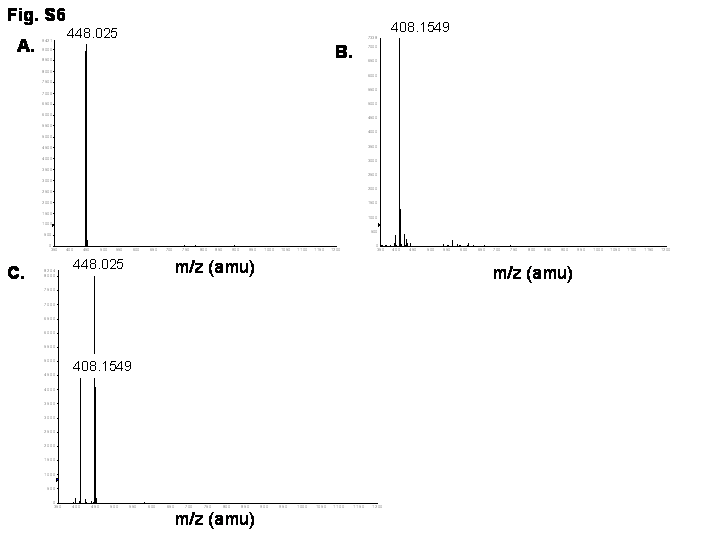

Supplement: Figure S6 — H-89 and coelenterazine-h do not form a covalent adduct. All samples were diluted in 50% ACN, 0.1% FA, loaded into electrospray needle, sprayed at 900 V, and detected from m/z between 350–1200. A) spectra of H-89. B) Spectra of coelenterazine-h. C) spectra of 1:1, H-89:coelenterazine-h (0.04 MB TIF) [file pone.0005642.s006.tif]
